# Murine bone-derived mesenchymal stem cells undergo molecular changes after a single passage in culture

**DOI:** 10.1038/s41598-024-63009-8

**Published:** 2024-05-29

**Authors:** Anastasia M. Hughes, Vincent Kuek, Joyce Oommen, Rishi S. Kotecha, Laurence C. Cheung

**Affiliations:** 1 Leukaemia Translational Research Laboratory, Telethon Kids Cancer Centre, Telethon Kids Institute, 15 Hospital Avenue, Nedlands, Perth, WA 6009 Australia; 2 Curtin Medical School, Curtin University, Kent Street, Bentley, Perth, WA 6102 Australia; 3 School of Biomedical Sciences, University of Western Australia, Perth, WA 6009 Australia; 4 UWA Medical School, University of Western Australia, Perth, WA 6009 Australia; 5 Department of Clinical Haematology, Oncology, Blood and Marrow Transplantation, Perth Children’s Hospital, Perth, WA 6009 Australia; 6 Curtin Health Innovation Research Institute, Curtin University, Kent Street, Bentley, Perth, WA 6102 Australia

**Keywords:** Differentiation, Stem cells, Developmental biology, Stem cells, Cell biology

## Abstract

The rarity of the mesenchymal stem cell (MSC) population poses a significant challenge for MSC research. Therefore, these cells are often expanded in vitro, prior to use. However, long-term culture has been shown to alter primary MSC properties. Additionally, early passage primary MSCs in culture are often assumed to represent the primary MSC population in situ, however, little research has been done to support this. Here, we compared the transcriptomic profiles of murine MSCs freshly isolated from the bone marrow to those that had been expanded in culture for 10 days. We identified that a single passage in culture extensively altered MSC molecular signatures associated with cell cycling, differentiation and immune response. These findings indicate the critical importance of the MSC source, highlighting the need for optimization of culture conditions to minimize the impact on MSC biology and a transition towards in vivo methodologies for the study of MSC function.

## Introduction

Mesenchymal stem cells (MSCs) are self-renewing, multipotent stem cells that can differentiate into bone, cartilage and adipose tissue. MSCs also regulate many physiological processes including hematopoiesis, angiogenesis and inflammation via secretion of cytokines, extracellular vesicles and cell–cell contact mechanisms^1–4^. Depletion or dysfunction of the MSC population has consequently been associated with many diseases^5^. A key avenue being explored is whether the differentiation potential, cytokine secretion and immunomodulatory function of MSCs can be applied in regenerative medicine^6,7^, with administration of MSCs showing promise for the treatment of burns, diabetic wounds and acute lung injury^8–10^.

However, the rarity of the MSC population poses an ongoing limitation for preclinical and clinical application. Although MSCs can be extracted from tissue types such as skin, adipose tissue, umbilical cord and bone marrow (BM), they are present at very low frequencies^11^. MSCs account for only 0.01–0.001% of BM mononuclear cells and therefore, expansion of MSCs in vitro is required prior to clinical or laboratory use^12^. Long-term culture can however, alter MSC morphology, proliferation, transcriptome and function, thus raising concern that MSC expansion in vitro may impair therapeutic efficacy^12–18^. How cell culture impacts MSC biology and how this can be mitigated remains unclear.

While many studies have compared the properties of early and late-passage MSCs, an understanding of how primary MSCs differ to those that have been cultured remains lacking^13–18^. Here, we compared freshly isolated bone-derived MSCs (primary MSCs) to bone-derived MSCs that underwent a single passage in culture (cultured MSCs), a direct comparison that has not been undertaken in MSC research. We utilized the protocol from Houlihan et al. to isolate MSCs based on the expression of Sca-1 and PDGFRα^19^. This study reported that isolating MSCs based on Sca-1 and PDGFR-α expression avoids hematopoietic contamination, and that MSCs were highly proliferative and almost without senescence in vitro. After 10 days in culture, we identified extensive transcriptomic alterations between the two groups. Genes upregulated in cultured MSCs were associated with cell cycle, DNA replication and mitosis, while downregulated genes were associated with an impairment to MSC differentiation and immunomodulatory capabilities. Overall, our results shed light on biological differences between primary MSCs and cultured MSCs which could have implications for their application.

## Results

### A single passage of MSCs in vitro alters the expression of genes associated with essential biological processes

**Figure 1:**
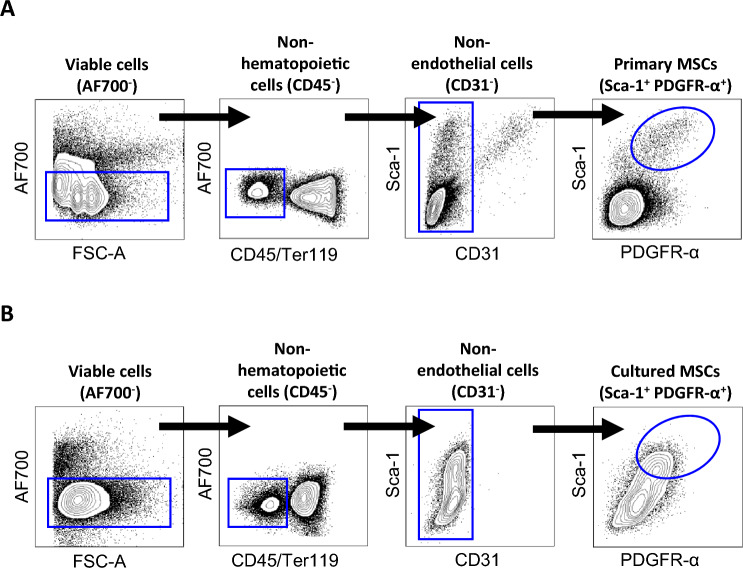
Gating strategy for MSC sorting. Viable (**A**) primary MSCs and (**B**) cultured MSCs were sorted based on cell surface markers CD45^-^Ter119^-^CD31^-^Sca-1^+^PDGFR-α^+^.

**Figure 2:**
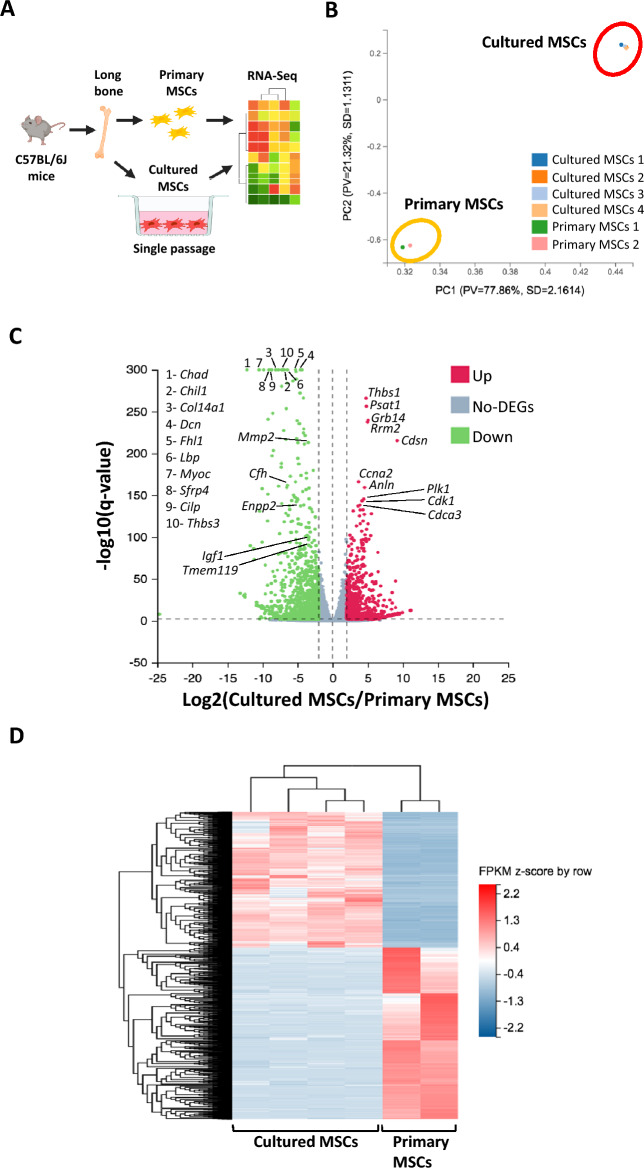
RNA sequencing reveals that cultured MSCs and primary MSCs are transcriptionally distinct. (**A**) Schematic of the experimental process to obtain cultured MSCs and primary MSCs for RNA sequencing. Schematic was generated by BioRender.com. (**B**) Principal component analysis based on row z-score of FPKM values for cultured MSC (n = 4) and primary MSC (n = 2) samples. (**C**) Volcano plot demonstrating the presence of 2165 differentially expressed genes (DEGs) in cultured MSCs compared to primary MSCs (|Log2FC|≥ 2, q-value ≤ 0.01). The top 10 most significantly upregulated DEGs and 15 selected DEGs that are significantly downregulated (according to q-value) are listed. Green represents downregulated genes; red represents upregulated genes. (**D**) Unsupervised clustering of DEGs as a heatmap. Heatmap is of FPKM values standardized by row z-score.

Fluorescence-activated cell sorting was used to isolate primary MSCs and cultured MSCs and RNA sequencing was performed to identify transcriptional profiles (Figs. 1, 2A). To assess variance within the dataset, we performed principal component analysis which identified distinct clustering between primary MSCs and cultured MSCs (Fig. 2B). Differential expression analysis identified a total of 2165 differentially expressed genes (DEGs) in cultured MSCs when compared to primary MSCs (|Log2FC|≥ 2, q-value ≤ 0.01). Of those genes, 952 were upregulated and 1213 were downregulated (Figs. 2C, D).

**Figure 3:**
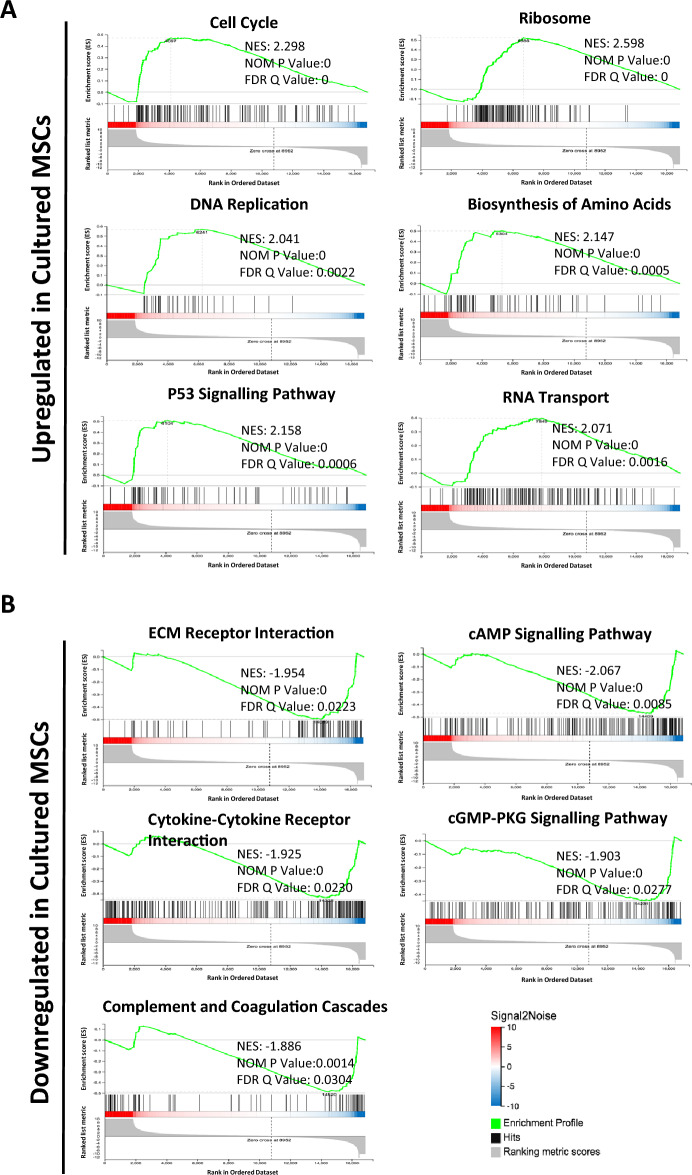
Gene set enrichment analysis of cultured MSCs and primary MSCs. Gene set enrichment analysis (GSEA) using the Kyoto Encyclopedia of Genes and Genomes (KEGG) database demonstrating **(A)** Gene sets upregulated in cultured MSCs and **(B)** Gene sets downregulated in cultured MSCs. For GSEA, a |normalized enrichment score (NES)|≥ 1, nominal (NOM) p-value ≤ 0.05 and false discovery rate (FDR) q-value ≤ 0.25 were used as threshold values.

To understand the potential biological ramifications of this altered gene expression we performed gene set enrichment analysis (GSEA). Genes sets associated with the cell cycle, DNA replication, P53 signalling pathways and protein synthesis pathways (i.e., ribosome, biosynthesis of amino acids, RNA transport) were significantly enriched in cultured MSCs (Fig. 3A). Conversely, downregulated genes were enriched for processes including extracellular matrix (ECM) receptor interactions, cytokine-cytokine receptor signalling, and the complement and coagulation cascades, which are the essential components of the innate immune response (Fig. 3B). Furthermore, the cAMP and cGMP signalling pathways, which are important for mediating a range of cellular responses to external stimuli, were also downregulated in cultured MSCs (Fig. 3B). Collectively, this indicates that the alterations to gene expression in cultured MSCs impacts on a number of essential biological processes.

### Gene ontology enrichment analysis reveals that genes upregulated in cultured MSCs are associated with cell cycle and cell division

**Figure 4:**
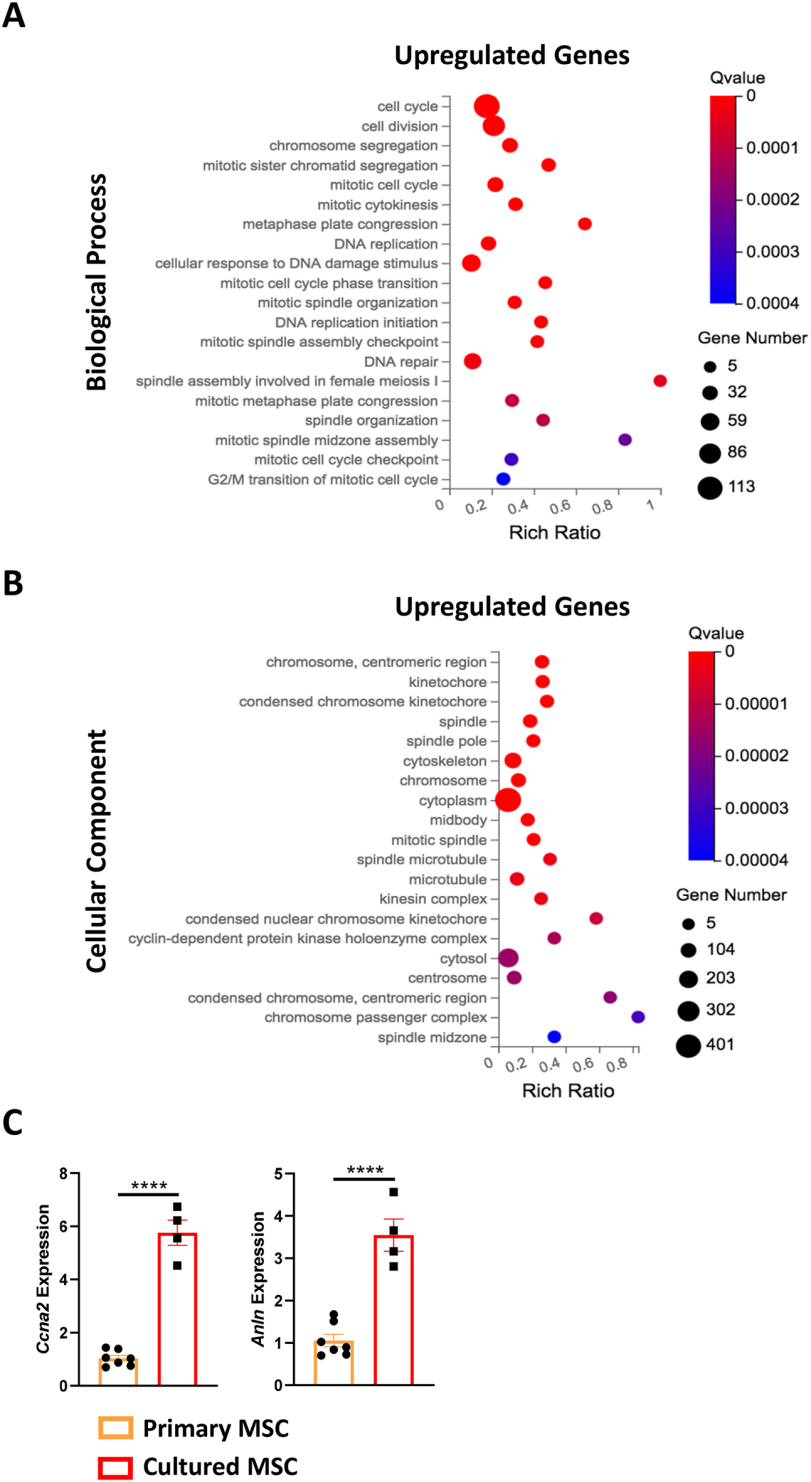
Gene ontology enrichment analysis and RT-qPCR validation of the upregulated genes in cultured MSCs compared to primary MSCs. (**A**) Gene ontology (GO) analysis of biological processes enriched in the 952 genes upregulated in cultured MSCs. The top 20 significantly enriched biological processes are displayed (based on q-value ≤ 0.05). (**B**) GO analysis of cellular components enriched in the 952 genes upregulated in cultured MSCs. The top 20 significantly enriched cellular components are displayed. (**C**) Upregulation of *Ccna2* and *Anln* genes as validated by RT-qPCR analysis. All datapoints are presented as biological replicates (n = 7 for primary MSCs and n = 4 for cultured MSCs). Data presented as mean ± SEM relative to primary MSCs. ****p ≤ 0.0001.

Further insight into the functional profile of cultured MSCs was gained by performing gene ontology (GO) enrichment analysis. Genes observed to be upregulated in cultured MSCs, such as cyclin A2 (*Ccna2*) (Log2FC = 3.69) and anillin (*Anln*) (Log2FC = 4.53), were enriched for biological processes including the ‘cell cycle’ and ‘cell division’ (Fig. 4A). Furthermore, processes such as ‘chromosome segregation’, ‘mitotic sister chromatid segregation’, ‘mitotic cell cycle’ and ‘mitotic cytokinesis’, were also enriched in upregulated DEGs. In line with these findings, upregulated genes were associated with nuclear cellular components such as the ‘chromosome’, ‘centromeric region’, ‘kinetochore’ and ‘spindle’ (Fig. 4B). The upregulation of *Ccna2* and *Anln* by MSCs following culture was further confirmed by quantitative reverse transcription polymerase chain reaction (RT-qPCR) (Fig. 4C). Taken together, we identified a clear transcriptomic enrichment for cell cycling and DNA replication-related processes.

### Gene ontology enrichment analysis reveals that genes downregulated in cultured MSCs are associated with differentiation and immune responses

**Figure 5:**
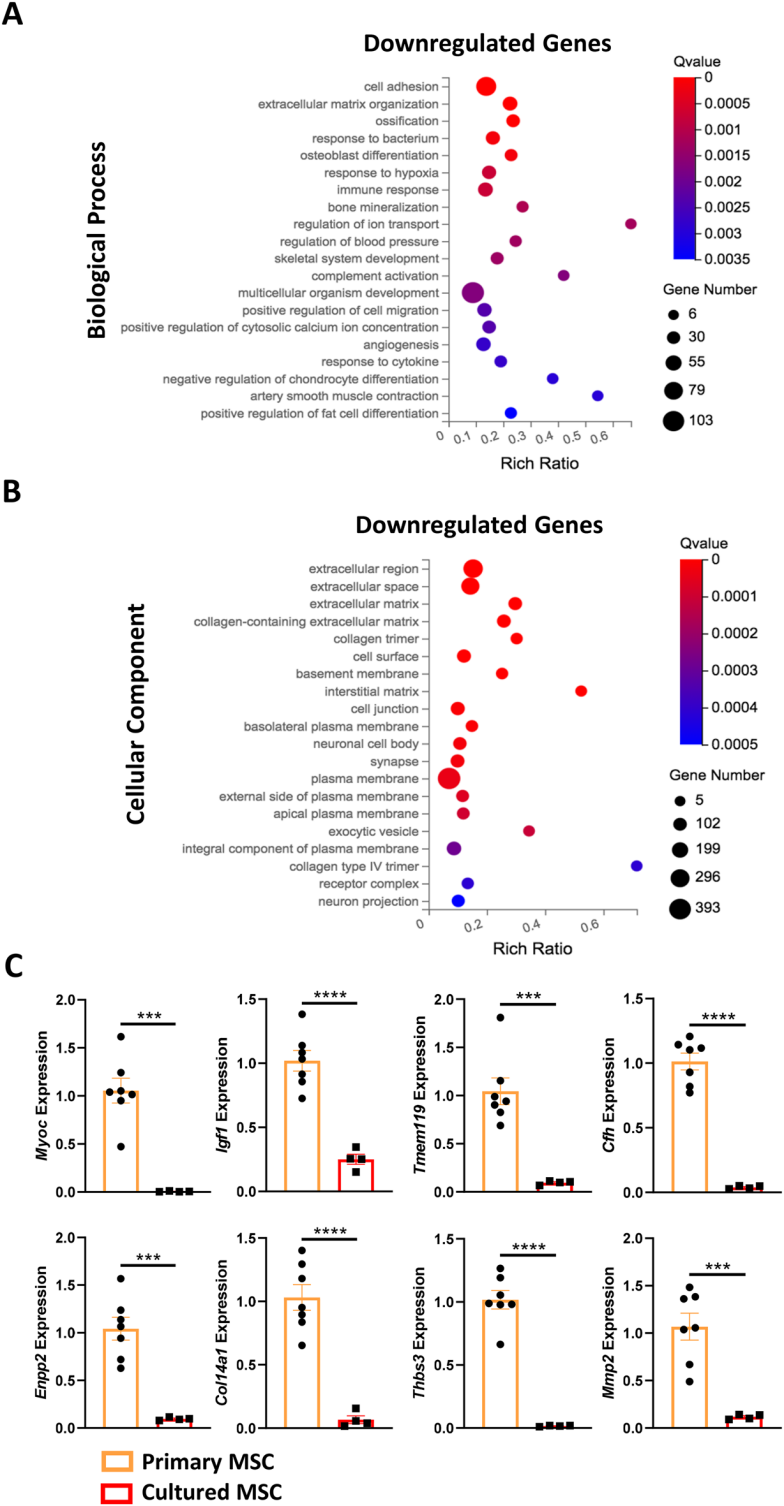
Gene ontology enrichment analysis and RT-qPCR validation of the downregulated genes in cultured MSCs compared to primary MSCs. (**A**) Gene ontology (GO) analysis of biological processes enriched in the 1213 genes downregulated in cultured MSCs. The top 20 significantly enriched biological processes are displayed (based on q-value ≤ 0.05). (**B**) GO analysis of cellular components enriched in the 1213 genes downregulated in cultured MSCs. The top 20 significantly enriched cellular components are displayed. (**C**) Downregulation of *Myoc*, *Igf1*, *Tmem119*, *Cfh*, *Enpp2*, *Col14a1*, *Thbs3* and *Mmp2* genes as validated by RT-qPCR analysis. All datapoints are presented as biological replicates (n = 7 for primary MSCs and n = 4 for cultured MSCs). Data presented as mean ± SEM relative to primary MSCs. ***p ≤ 0.001, ****p ≤ 0.0001.

GO enrichment analysis of genes downregulated in cultured MSCs identified an enrichment for biological processes that are essential for physiological MSC function. For instance, genes associated with ‘osteoblast differentiation’, including myocilin (*Myoc*) (Log2FC =  − 10.46), insulin-like growth factor 1 (*Igf1*) (Log2FC =  − 3.44) and transmembrane protein 119 (*Tmem119*) (Log2FC =  − 3.44), were downregulated in cultured MSCs (Figs. 2C, 5A). Likewise, genes associated with ‘ossification’ and ‘bone mineralization’ were also downregulated in cultured MSCs, implying that cultured MSCs possess impaired osteogenic potential (Fig. 5A). Similarly, genes associated with promoting adipocyte differentiation (‘positive regulation of fat cell differentiation’) were also downregulated in cultured MSCs. In contrast, genes involved in the ‘negative regulation of chondrocyte differentiation’ were downregulated, demonstrating a potential bias towards the chondrogenic lineage in cultured MSCs (Fig. 5A). Overall, a clear perturbation to cultured MSC differentiation potential is evident at the molecular level.

Another essential function performed by MSCs is modulation of the immune response via interactions with B cells, T cells, natural killer cells and dendritic cells^1^. GO enrichment analysis identified that ‘immune response’ and ‘complement activation’ processes were downregulated in cultured MSCs. Specifically, cultured MSCs exhibited decreased expression of genes such as complement factor H (*Cfh*) (Log2FC =  − 6.45) and ectonucleotide pyrophosphatase/phosphodiesterase 2 (*Enpp2*) (Log2FC =  − 4.59) (Fig. 2C). These results suggest that the immunomodulatory capabilities of MSCs may also be impaired following a single passage in vitro.

Finally, GO enrichment analysis of cellular components identified that downregulated genes were associated with the ‘extracellular region’, ‘extracellular space’, ‘cell surface’ and ‘plasma membrane’ (Fig. 5B). In addition, GO enrichment analysis identified enrichment of biological processes including ‘cell adhesion’ and ‘ECM organisation’, demonstrating that downregulated genes are important for intercellular interaction and regulating the ECM (Fig. 5A). Specifically, genes mediating cell–cell adhesion such as collagen type XIV alpha 1 (*Col14a1*) (Log2FC =  − 8.11) and thrombospondin 3 (*Thbs3*) (Log2FC =  − 7.24) were downregulated in cultured MSCs (Fig. 2C). Matrix metalloproteinase 2 (*Mmp2*) (Log2FC =  − 3.51), which is responsible for ECM breakdown and remodelling was also downregulated in cultured MSCs (Fig. 2C). Downregulation of these genes in cultured MSCs compared to primary MSCs was confirmed by RT-qPCR (Fig. 5C). These findings suggest alterations to the mechanisms by which MSC’s interact with the microenvironment following a single passage in vitro.

## Discussion

Over the years, extensive research has established the role of MSCs in diseases such as cancer^20,21^. MSC-based regenerative therapies have been trialled and implemented for a range of injuries and illnesses^11,22^. However, the rarity of this stem cell population necessitates its expansion in culture prior to clinical use. The most commonly used passages for transplantation are from passage 3 to 7, however many studies have found that the passaging process inadvertently alters MSC biology and function^13–18^. To date, changes identified by culturing MSCs at increasing passages include alterations to DNA damage response, senescence, multipotent potential and immunomodulatory properties^13–18^. Others have shown that an increase in the passage number does not significantly impact the immunoprivilege of MSCs^23^. However, no study has directly compared changes between primary MSCs and MSCs after one passage. Here we provide unique insight into culture-induced molecular changes in MSCs following just a single passage.

Previous studies have demonstrated that MSCs have limited proliferative potential in vitro and undergo senescence with increasing cell passages^13,15^. However, it should be noted that these studies examined the biological aging of MSCs when the cells were already under maintenance in an in vitro setting. In contrast, our study explored the molecular impact of cell culture on MSCs using primary MSCs and cultured MSCs. In fact, we observed a clear distinction in the molecular signatures of cultured MSCs and primary MSCs, particularly in upregulation of genes involved in ‘cell cycle’, ‘cell division’ and ‘DNA replication’ in cultured MSCs, suggesting that MSC turnover is increased following early passage in vitro. Our results identified that just 10 days in culture induced the upregulation of genes associated with cell cycling. *Ccna2* and *Anln* are responsible for cellular proliferation and were the most significantly upregulated genes associated with ‘cell cycle’ and ‘cell division’ biological processes^24,25^. In support of this, we also found that the number of the cultured MSCs after 10 days in culture (average of 1,03,250 cells per mouse) was approximately 27-fold higher than the number of the primary MSCs (average of 3850 cells per mouse). Further studies should be conducted to further investigate the mechanistic role of these genes in this setting and exploit the strategy of genetically manipulating *Ccna2* and *Anln* in vitro to overcome MSC senescence with increasing cell passages. In addition, western blot analysis could also be conducted to evaluate changes in the signaling molecules associated with cell cycle and cell division, such as cyclins, PCNA and Ki67.

Another important characteristic of MSCs is their trilineage differentiation potential. Whilst we previously confirmed that cultured MSCs can differentiate into osteoblasts, adipocytes and chondrocytes, our transcriptomic analyses suggest that differentiation potential may vary between primary MSCs and cultured MSCs^26^. For example, GO enrichment analysis identified that genes associated with ‘osteoblast differentiation’, ‘ossification’ and ‘bone mineralization’ were downregulated in cultured MSCs. A reduction in MSC osteogenic potential has previously been noted following long-term culture^15,17^. However, our data provide striking evidence that the osteogenic potential of MSCs may be compromised as early as following a single passage in culture. Interestingly, one study has successfully demonstrated the feasibility of preconditioning MSCs with cytokines (e.g., tumor necrosis factor-alpha) to promote osteogenesis^27^. These findings are clinically important as MSCs are being trialled for their potential to regenerate bone in human subjects^28^.

GO enrichment analysis also identified changes to the adipogenic- and chondrogenic-lineage potentials of MSCs following culture. While contention remains regarding the effect of long-term culture on the adipogenic potential of MSCs, our data suggest that culturing MSCs could potentially impair adipogenesis after just one passage^15,17,29^. Furthermore, genes classified as negative regulators of chondrocyte differentiation were downregulated in cultured MSCs, indicating a possible differentiation bias towards the chondrocyte lineage. However, others have reported reduced chondrogenic potential in MSCs following long-term culture, indicating that any bias towards the chondrocyte lineage may only be short-lived^29^. Future studies could validate the effect of cell culture on the differentiation potential of primary MSCs by performing tri-lineage differentiation assays. In addition, the self-renewal capacity of MSCs could also be investigated by comparing the colony-forming unit ability of primary MSCs and cultured MSCs.

MSCs are also capable of regulating the proliferation and activation of immune cells, thereby modulating the body’s response to injury and illness^30,31^. Many studies are currently exploring the role of MSC immunomodulation in disease and for the treatment of immune disorders^32^. However, long-term culture can impair the immunosuppressive capabilities of MSCs^18^. Corroborating these findings, we identified that genes downregulated in cultured MSCs were enriched for biological processes including ‘response to bacterium’, ‘immune response’ and ‘complement activation’, indicating that impairment of the immunomodulatory ability of MSCs may be altered after just 10 days in culture. For example, a gene found to be downregulated in cultured MSCs was *Enpp2,* which encodes for autotaxin. Autotaxin is known to mediate lymphocyte migration, thus supporting the proposition that its downregulation may impair MSC immunomodulation^33^. Future studies could validate the immune modulatory properties of primary MSCs and cultured MSCs by priming these cells with cytokines such as IFN-γ, TNF-α, and IL-1β, and subsequently assess changes in the production/secretion of functional factors that regulate immune responses.

Interestingly, the impact of cell culture on the molecular signature of MSCs have also been demonstrated in several studies involving the use of MSCs derived from human bone marrow. For instance, genes involved in regulation of the complement cascade (*SERPING1*), antitumoral immunity (*OAS2*) and osteogenesis (*ALPL*) were found to be downregulated in cultured adherent human bone marrow-derived MSCs compared to primary human bone marrow-derived MSCs^34^. In addition, separate studies have shown that uncultured human bone marrow-derived CD45^low^CD271^+^ MSCs demonstrate distinct transcriptional differences compared to cultured bone marrow-derived MSCs^35,36^. These data from primary human bone marrow MSCs corroborate our findings from murine bone-derived MSCs, supporting the impact of culture-induced molecular changes on MSCs.

Another area of interest is the impact of culture methodologies on MSC biology. Unlike human bone marrow, murine MSCs are not easily isolated from bone marrow aspirates by plastic adherence due to contaminating hematopietic cells. However, the discovery that MSCs are found lining the inner surface of compact bones has given rise to a new method where murine MSCs can be directly isolated from bones cleared of bone marrow, including one pioneered by Zhu et al. where MSCs were successfully isolated from collagenase II-digested bone fragments in vitro^37^. Although this method can remove contaminating hematopoietic cells, it is worth noting that this would involve multiple cell passages which could inadvertently affect MSC biology. Therefore, this necessitates further protocol refinements in the future. In addition, several studies have examined the influence of technical variables including culture medium, dynamic culture, 3-dimensional (3D) culture and oxygenation on MSC biology^38–45^. For instance, a previous study has shown that high levels of oxygen exposure can alter multilineage differentiation capacity of primary bone marrow-derived mouse MSCs, as well as inducing cellular stress, growth arrest and apoptosis via p53 activation^46^. Whilst our study did not specifically examine whether atmospheric oxygen can affect the physiology of long bone-derived MSCs and p53 activity, this remains an avenue for future investigation. Remarkably, dynamic culture systems, preconditioning with cytokines and the use of 3D scaffolds has been found to improve the osteogenic differentiation potential and/or immunomodulatory capabilities of cultured MSCs, which highlights the importance of optimizing culture methodology^41,47–49^. It is also worth noting that there is a lack of consensus with regards to defining MSCs based on surface markers. For instance, some studies define populations of MSCs based on the expression of LEPR and Nestin, while we defined our bone-derived MSCs based on Sca-1 and PDGFRα^19,50,51^. Therefore, consensus regarding a standardized protocol for MSC definition and culture will be beneficial for stem cell research going forward.

A key limitation of our current study is the difficulty of obtaining high numbers of primary MSCs for bulk RNA sequencing via fluorescence-activated cell sorting (FACS) due to the rarity of this population. While we managed to obtain a total of four cultured MSC samples from culturing heterogenous bone cells prior to FACS, we sorted and pooled primary MSCs from four mice and six mice respectively, which only yielded two biological samples for the primary MSC group. Future studies could utilize next-generation sequencing techniques, such as single cell RNA sequencing to overcome this limitation. It is also important to note that our study can only delineate the molecular mechanisms, rather than biological properties using conventional assays due to the fundamental difference between in situ-derived primary MSCs and in vitro-derived cultured MSCs. These differences may also be reflected by the notion that MSCs reside in BM niches where crosstalk among MSCs and niche cells influence their biology, in contrast to mono-cultured MSCs which are deprived of this critical influence. Preclinical experiments which utilize cultured MSCs may therefore not accurately recapitulate in vivo phenotypes and thus consideration of this caveat should be made when interpreting results.

In conclusion, this study provides insight into the molecular changes of murine MSCs after a single passage in culture. Our findings provide a foundation from which we can optimize MSC culture methodology to maintain the characteristics of primary MSCs and investigate the impact of culture-induced changes on the therapeutic efficacy of MSCs in the clinic.

## Methods

### Isolation and staining of mesenchymal stem cells

Ten-week-old female C57BL/6J mice were euthanized by isoflurane followed by cervical dislocation. All animal experiments reported in this study were conducted in accordance with ARRIVE guidelines, and all other experiments were performed in accordance with relevant guidelines and regulations. Long bones (tibias and femurs) were excised and bone marrow cells were flushed out and discarded. Bones were then fragmented, followed by enzymatic digestion with 1.5 mg/ml collagenase type IV (Worthington biochemical corp.) and 0.1 mg/ml DNase I (Sigma–Aldrich) at 37 °C for 60 min under mechanical agitation, in accordance with previously published protocols^26^. Bone digests were then cleared of bone fragments via filtration with 100 μm cell strainers (Corning) to obtain a mixed cell population in suspension. The heterogenous bone cell population was resuspended in 5% fetal calf serum in phosphate buffered saline (FCS/PBS). To obtain primary mesenchymal stem cells (MSCs), cells were stained with BD horizon fixable viability stain 700 (BD biosciences) to exclude non-viable cells. This was followed by staining with mouse CD45-PerCP-Cy5.5 and Ter119-PerCP-Cy5.5 antibodies to exclude hematopoietic cells, and mouse CD31-FITC antibody to exclude endothelial cells. Primary MSCs were then identified by staining with mouse Sca-1-BV510 and PDGFRα-APC antibodies, and isolated using fluorescence-activated cell sorting (FACS)^52^. To obtain cultured MSCs, the heterogenous bone cell population was first cultured in MesenCult^™^ basal medium (#05,514, stem cell technologies) supplemented with 100 Units/ml penicillin, 100 μg/ml streptomycin (Thermo Fisher Scientific), 1% l-glutamine (Thermo Fisher Scientific), 10% MesenCult^™^ supplement (#05515, stem cell technologies) and 0.1% MesenPure^™^ (#05500, stem cell technologies) for 10 days in T25 flasks (Thermo Fisher Scientific). Adherent cells were then washed with PBS and enzymatically detached via TrypLE™ express enzyme (Thermo Fisher Scientific). Cells were then resuspended in 5% FCS/PBS, followed by staining with the same antibodies to identify and sort the cultured MSCs. All antibodies for flow cytometry were obtained from BD Biosciences, except for PDGFRα-APC (eBioscience). Following isolation by FACS, both the primary MSCs and cultured MSCs were lysed with RLT lysis buffer (Qiagen) and stored at − 80 °C until RNA extraction. The gating strategy for the isolation of primary MSCs and cultured MSCs is shown in Fig. 1.

### RNA sequencing of MSCs

For RNA extraction and sequencing, we pooled 12,000 cells from four mice and 28,000 cells from six mice to yield two biological replicates of primary MSCs. For cultured MSCs, we obtained four biological replicates ranging from 1,35,000–2,74,000 cells; each replicate was derived from two mice following culture. RNA sequencing was performed in accordance with previously published protocols^26^. In brief, total RNA was extracted from cell lysates using a RNeasy micro kit (Qiagen). The quality and quantity of RNA samples was determined using an Agilent 2100 bioanalyzer to confirm an acceptable RNA integrity number (> 6.5). Samples were then sent to BGI genomics, Hong Kong for sequencing and data pre-processing. RNA amplification and cDNA production was conducted using the SMART-Seq v4 ultra low input RNA kit (Takara Bio), utilizing the same amount of input RNA from each sample. Quality control and circularization of amplified products was performed, followed by 100 bp paired-end RNA sequencing using the BGISEQ-500 platform.

All data were processed and analyzed using Dr Tom software (BGI Genomics https://biosys.bgi.com/#/report/login), in accordance with previously published methods^26^. For quality control, reads containing the adaptor, unknown base N content greater than 5% and low-quality reads were removed from raw data using SOAPnuke software (v1.5.2, BGI). Clean reads (58–71 million/sample) were aligned to the reference genome (Mus_musculus, NCBI, version: GCF_000001635.26_GRCm38.p6) using HISAT2 (v2.0.4). Alignment to reference gene was conducted using Bowtie2 (v2.2.5). The gene expression level for each sample was calculated using RSEM (v1.2.8). Differentially expressed genes were defined (Log2FC|≥ 2, q-value ≤ 0.01) and calculated using the DEseq2 method. Principle component analysis, gene ontology enrichment analysis and gene set enrichment analysis (GSEA) were performed using BGI’s Dr Tom software as previously described^26^. For GSEA analysis, a |normalized enrichment score|≥ 1, nominal p-value ≤ 0.05 and false discovery rate q-value ≤ 0.25 were used as threshold values. Expression levels for all genes were normalized as fragments per kilobase of transcript per million fragments mapped (FPKM), which accounts for sequencing depth and gene length. Sequencing data were uploaded and are available in the gene expression omnibus (GEO) database under the accession number GSE240514.

### Quantitative reverse transcription polymerase chain reaction

For RT-qPCR, isolation and extraction of RNA was performed as described above. Biological replicates from seven mice for primary MSCs and from four mice following culture for cultured MSCs were obtained. cDNA was generated using SuperScript VILO master mix. To perform quantitative reverse transcription polymerase chain reaction (RT-qPCR), cDNA was prepared in a reaction mix of Taqman fast advanced master mix and the following TaqMan gene expression assays: *Ccna2 (*Mm00438063_m1), *Anln* (Mm00503748_m1), *Myoc* (Mm00447900_m1), *Igf1* (Mm00439560_m1), *Tmem119* (Mm00525305_m1), *Cfh* (Mm01299248_m1), *Enpp2* (Mm00516572_m1), *Col14a1* (Mm00805269_m1), *Thbs3* (Mm00449802_m1), *Mmp2* (Mm00439498_m) and *Hprt* (Mm03024075_m1). The CT value for each individual sample was normalized to *Hprt* and relative expression levels were calculated using the ∆∆CT method^53^. All reagents for cDNA synthesis and RT-qPCR reactions were obtained from Thermo Fisher Scientific. RT-qPCR was performed using QuantStudio 7 Flex real-time PCR system (Applied Biosystems).

### Statistical analysis

Statistical analyses were performed using Prism version 8.1.1 (Graphpad). For comparison between two groups, data were analyzed using the two-tailed unpaired student’s *t*-test. A p-value of ≤ 0.05 was considered statistically significant.

### Experimental model and study participants details

Ten-week old female C57BL/6J mice were obtained from the Animal Research Centre, Perth and housed under pathogen-free conditions. Mice were housed at 21–22 °C with 12 h light/dark cycle. All animal work conducted in this study was approved by the Telethon Kids Institute Animal Ethics Committee (AEC), under the following approval numbers and titles:

AEC#330: investigating the interaction between bone cells and leukaemia cells (approved 16/10/2017).

AEC#P2176: investigate the crosstalk between bone cells and leukaemia cells (approved 01/09/2022).

## Data Availability

RNA-sequencing data presented in this study can be found in the Gene Expression Omnibus database under accession number GSE240514.
